# Proceedings of Medical Societies

**Published:** 1868-01-01

**Authors:** 


Proceedings of Medical Societies.—Thanks are returned to friends who have forwarded memoranda of the transactions of their local organizations. That these have not been published in the Journal has not been from want of proper appreciation of their kindness, or deficiency in respect, but simply from physical impossibility. Since the present Editor has been in charge of the Journal, the number of these communications received has been such, that to have published them would have absolutely excluded all other matter. Abstracts of valuable papers, or the papers in full, if not too lengthy, will be cheerfully inserted whenever opportunity offers.

